# Editorial: Heavy Metal Toxicity in Plants: Recent Insights on Physiological and Molecular Aspects

**DOI:** 10.3389/fpls.2021.830682

**Published:** 2022-02-18

**Authors:** Rafaqat Ali Gill, Mukesh Kumar Kanwar, Andre Rodrigues dos Reis, Basharat Ali

**Affiliations:** ^1^Key Laboratory of Biology and Genetic Improvement of Oil Crops, The Ministry of Agriculture and Rural Affairs, Oil Crops Research Institute of Chinese Academy of Agricultural Sciences, Wuhan, China; ^2^Department of Horticulture, College of Agriculture and Biotechnology, Zhejiang University, Hangzhou, China; ^3^São Paulo State University “Júlio de Mesquita Filho” (UNESP), Tupã, Brazil; ^4^Department of Agronomy, University of Agriculture, Faisalabad, Pakistan

**Keywords:** heavy metals stress, plant genome, phytoremediation, nutrient deprivation, management, cell morphology

The topmost soil (i.e., surface O and A) is of great importance for plants as it contains the majority of the mineral elements required for normal plant growth and development (PG&D). However, the same soil also serves as a sink for hazardous pollutants including heavy metals (HMs) such as cadmium (Cd), lead (Pb), chromium (Cr), arsenic (As), and mercury (Hg) concentrated due to anthropogenic activities (industrialization and urbanization) that severely impact on PG&D. These toxic metal ions after entering into plant body disturbed cell wall, cell membrane, the activity of mitochondria (root) and cytoplasm (leaf) induced MDA and ROS content, and negatively impact the antioxidative machinery, fatty acids (i.e., linoleic acid and linolenic acid), and some other enzymes such as NADPH oxidase, etc. Further, HM toxicity caused the disturbance of water and nutrient supply from soil to upper parts of plants and deterioration in the activities of leaf pigment such as chlorophyll content and PS II impact on the sugar production and then transportation system from upper to lower parts of plants. These above drastic changes altogether caused the alterations in the various physiological and biochemical processes resulted in the reduction of the fresh biomass (root and shoot), overall PG&D, and finally decreased the grain quantity and quality. To tackle this, the researchers explored various perspectives while studying plants grown under HMs stressed environments. First, they explored plant response mechanisms as they activate its enzymatic (SOD, POD, CAT, APX, and GR) and non-enzymatic (GSH and ASA) antioxidant machinery. Secondly, plants modify their cell wall and root exudates. The third strategy is the utilization of plants (phytoremediation-hyperaccumulation) to absorb more content of HMs from the soil. Fourth, overexpression of metal responsive genes such as *PCR2, AT6, CDT1*, and *WRKY* creates the protective shield against metal toxicity. Lastly, exogenous applications of growth regulators such as GA, H_2_S, and GSH provide relief to plant species coping hazardous behavior of HMs. In a nutshell, an integrated strategy based on above all alternatives can strengthen the plant defense system and increase the seed yield of the crop plants growing in the toxic environment.

This Research Topic primarily focuses on HM induced abnormalities in plants, like nutrient imbalance, alteration in PG&D, microflora degradation, and enzymes dis-functioning. The present “Research Topic” succeeded in collecting 11 original research papers, one review and one perspective on diverse HMs like Cd, Pb, zinc (Zn), Cr, nickel (Ni), aluminum (Al), and a signaling molecule hydrogen sulfide (H_2_S).

## Cd Toxicity in Plants: Hazardous Impacts and Its Alleviation Through Various Strategies

Cadmium (Cd) becomes a part of the soil–plant environment as plants absorbs water and nutrients from the soil. In this context, Shi et al. explore the alleviating role of potassium (K) fertilizers to reduce the Cd content in *Panax notoginseng* by enhancing activities of soil micro-organism as they influenced by soil pH, total organic matter (TOM), and cation exchange capacity (CEC). Their pot experiment's results probed the impact of exogenously applied K on the biologically available of amount of Cd content (bio-Cd), soil pH, TOM, CEC, and endogenous Cd content in *Panax notoginseng*. Moreover, they considered that K_2_SO_4_ (0.6 g·kg^–1^) was optimal treatment to reduce the Cd uptake. However, in the field condition their results showed the positive impact of K_2_SO_4_ on the soil microbial community (i.e., Acidobacteria, Mortierellomycota, Proteobacteria, and Bacteroidetes) and the consequences on the soil pH, TOM, and CEC. Potassium application enhances the activities of Mortierellomycota, Proteobacteria, and Bacteroidetes and decreased the activity of Acidobacteria. Moreover, they explored the correlation of above-mentioned microbe species with soil pH, TOM, CEC, and bio-Cd. In a nutshell, K fertilizers decreased the accumulation of Acidobacteria, as they involved in the acidification of the soil pH and CEC, and enhanced the activities of Mortierellomycota, Proteobacteria, and Bacteroidetes, which were found to be responsible for increased pH, TOM, and CEC and reduced the bio-Cd content in the soil.

Oilseed rape (*B. napus*) contains soluble lipids including tocopherols, also known as vitamin E, is an essential nutrient for animals and human beings. The finding by Ali et al. suggested that tocopherols act as an antioxidant in tackling the reactive oxygen species (ROS) induced by Cd stress in two contrasting genotypes (Jiu-Er-13XI and Zheyou-50, differing in seed oil content) of *B. napus*. Their results showed that Cd significantly caused more damages in low seed oil content containing cultivar (Jiu-Er-13XI) compared to high seed oil content cultivar (Zheyou-50). They probed that level of ROS was significantly high in low seed oil content cultivar and physiological damages was appeared in terms of destroyed chloroplast and photosynthetic structures. Further, total fatty acids such as linoleic acid (18:2) and linolenic acid (18:3), were deteriorated due to increase in malondialdehyde (MDA) and tocopherols content, and Cd accumulation were more prominent in the roots of low seed oil content cultivar compared to high-seed oil content cultivar. They also showed that in resistant cultivar (Zheyou-50), the accumulation of α-Tocopherol and transcript levels of genes involved in the biosynthesis of tocopherol were much higher. As a whole, tocopherol not only restricts the uptake of Cd in roots and then translocation to the shoots but also scavenging ROS, lowering the MDA content, and play a role in polyunsaturated fatty acids remained intact with chloroplast membrane.

As an adaptive strategy, plants have evolved a diverse set of mechanisms to cope with Cd stress. Their strategy involved a combination of enzymatic, non-enzymatic antioxidants, extrusion of Cd across the plasma membrane, restriction of Cd movement toward roots and finally sequestration of Cd to the metabolically inactive plant parts such as root cell wall and leaf vacuoles. Recent progress on the role of WRKY transcription factors (TFs) (pronounced “worky” are the proteins that contain WRKY conserved domain and are involved in the regulation of several biotic and abiotic stress responses) in conferring the Cd tolerance in various plant species evidenced its role. Cai et al. have identified 29 Cd-responsive WRKY-TFs in Soybean. Among, 26 genes were upregulated and three were downregulated under Cd treatment. They identified a novel Cd-responsive GmWRKY142 gene and cloned to investigate its detailed tolerance mechanism in Cd stress. Their result showed that the above TF was localized in the nucleus and exhibit higher transcriptional activity in root when plants subjected to Cd stress. Moreover, in the overexpressed (*GmWRKY142*) lines (both in Arabidopsis and Soybean), expression levels of other Cd-responsive genes such as *ATCDT1, GmCDT1-2* encoding cadmium tolerance 1, and *GmCDT1-1* were enhanced compared to wild type (WT), which indicated that the upregulation of *GmCDT1-1* and *GmCDT1-2* resulted in the deterioration of Cd uptake and improves tolerance. Thus, GmWRKY142-GmCDT1-1/2 cascade opened up a new avenue/strategy to potentially cope Cd accumulation in Soyabean.

Another adaptive strategy to cope with Cd toxicity is by growing hyperaccumulators to the affected soils as they contained a sophisticated metal detoxification system. For example, in China *Sedum alfredii* Hance is a potential Cd hyperaccumulator plant as reported by Lin et al. They explored Cd-detoxification mechanism in hyperaccumulating ecotype (HE) of *Sedum alfredii* and discovered a gene named SaPCR2 that belongs to a plant Cd resistance (PCR) family. In the over-expressed lines, *SaPCR2* was highly expressed in root and its expression level not changed significantly under Cd exposure. Moreover, subcellular localization results in tobacco leaves and yeast showed that *SaPCR2* is a plasma membrane localized gene and its expression level (its protein in a Zn/Cd-sensitive yeast Δ*zrc1*) enhanced significantly resulted in the increased tolerance to Cd stress by deteriorating Cd content in cells. These findings suggested that overexpression of *SaPCR2* protects the root cells from Cd phytotoxicity by Cd leak out from the root cells. As a whole, these above findings suggested alternative routes to cope Cd tolerance and provides the basis for producing the metal tolerance commercial cultivars.

## Pb Toxicity in Plants: Negative Influence, and Antioxidant and Genomic Responses

Lead is considered as non-essential element and negatively affect the plant growth, when taken by plants. Huang et al. reveals the differences of tolerance between *M. azedarach* and *L. lucidum* under Pb–Zn polluted mine-tailings. Their results suggested that Pb-Zn pollutant significantly deteriorated the net photosynthetic rates and leaf photosynthetic pigment content by biochemical limitation (BL). Moreover, they explored that Pb-Zn tailing particularly affected on the PSII activity as evidenced the changes in the values of reaction center (RC) in the forms of electron movement, maximum quantum yield for primary photochemistry, the density of PSII RC per excited cross-section, and the absorption of antenna chlorophylls per PSII RC. Besides, they noted that Pb–Zn toxicity also affected the oxidation and reduction state of PSI resulted in an enhancement of ROS contents and lipid peroxidation. Further, their antioxidant activities related data showed that at lower level of Pb-Zn tailing catalase (CAT), superoxide dismutase (SOD), and peroxidase (POD) activities were increased but under higher level of Pb-Zn tailing were started declining. Both species showed the diversity in response to the above pollutants as *L. lucidum* exhibits more tolerance in terms of better net photosynthesis efficiency and higher enzymatic antioxidant activities. Another possible strategy to cope the Pb toxicity is the exogenous application of growth regulators. For instance, Khan M. et al. studied the negative impact of Pb stress and alleviating role of reduced glutathione (GSH) in cotton crop. Their controlled experiment suggested that Pb exposure (10 days) increased the MDA content and ROS level such as hydrogen peroxide (H_2_O_2_) and hydroxyl radical (OH•) and deteriorated the activities of CAT and ascorbate peroxidase (APX) in terminal and radial leaves. In contrast, CAT activity was enhanced and APX was decreased in the primary and secondary root. Similarly, activities of SOD and POD were increased in the median leaves and declining trend was recorded in the terminal leaves, primary and secondary roots. Moreover, glutathione reductase activity, ascorbic acid (ASA) and GSH contents were increased in all plant parts; except ASA content was deteriorated in the median leaves. Their electron microscopic observations from terminal and median leaves further evidenced the negative influence of Pb toxicity in the forms of damaged cell wall, cell membrane, chloroplast, and mitochondria. Similarly, in the root's micrographs, Pb induced damages were noticed in nucleus, cell membrane, and mitochondria. Interestingly, exogenously GSH application had significant impact on plants in terms of stabilized leaf and root cell ultra-structures by modulating the cell membranes, formation of multivesicular body vesicles and maintaining the structural integrity of other organelles. In a nutshell, GSH is involved in the alleviation of Pb-induced negative impacts in the cotton. Lastly, the review by Aslam et al. highlights the mechanism(s) involved in Pb uptake and translocation in cereals, critically reviews the possible management strategies to menace Pb toxicity in cereals. In detail, they have discussed that high Pb-stress caused the negative impacts on biochemical and physiological processes that are responsible for grain quality in cereals. Pb effect not only impact on the cell organelles integrity including membrane stability index but also several metabolic processes involved in the electron transport chain, PSII connectivity, mineral transportation, oxygen evolving complex and antioxidant system. Further, they argued on the role of plant growth-promoting rhizobacteria as potential and less expensive Pb-remediators in the soil. Lastly, they insight on the molecular and other cellular changes occurred by the utilization of TFs such as bZIP, ERF and GARP, and other proteins including MTP and NRAMP to cope the Pb induced toxicity.

## Cr Toxicity in Plants: Chelate-Assisted Phytoremediation

After Cd and Pb, Cr is considered the most harmful HM in the soil as it is present in the topsoil where plants struggle for the macro- and micro-nutrients to continue their normal life processing. To extract HMs from soil, utilization of chelators (chelator-assisted phytoremediation) is one of the most suitable strategy in terms of sustainability and economics as it enhance the bio-availability of HMs, which then increased the process of phytoextraction. Farid et al. study the role of organic chelator, glutamic acid (GA) in improving the phytoextraction of Cr by utilizing the sunflower (*H. annuus*). Their result first stated the negative role of Cr stress (with increasing concentration in the top soil) as it significantly reduced the plant growth and development resulted in deterioration in biomass, leaf areas antioxidant activities, and photosynthetic machinery related attributes. Secondly, their results showed that exogenously applied GA statistically improved the Cr-induced hazardous impacts. Interestingly, GA application increased the Cr uptake in the plants by enhancing availability and mobilization of Cr toward roots of sunflower plants that later on translocated to the upper plant's parts. As a whole, these finding suggested the dual role of GA as its application not only protects the plants form Cr-induced toxicity but also enhanced Cr accumulation (phytoextraction) by sunflower plants.

## Ni-Induced Toxicity in Plants: Impact on The Macro-Molecules, and Its Alleviation Through Hyper-Accumulation and Bio-Accumulation

Ni is an essential micro-element for higher plant species but required at very low concentrations. However, excess Ni in soil medium can be toxic and may disturb the normal PG&D. Growing evidence stated that like other HMs, extreme Ni stress also caused the production of ROS that severely damage the cell ultrastructure. For example, Khan F. et al. investigated negative impact of Ni induced toxicity alone and with deprivation of macro-nutrients i.e., nitrogen (N), phosphorus (P), and potassium (K) on nutrient uptake, plant growth, oxidative metabolism in rice seedlings. Their results stated that both Ni and nutrient deprivation induced stress severely negative impact on the establishment of rice seedlings, reduction in shoot length and fresh biomass. Among all combinations of Ni with N, P, and K, more severe results were recorded in Ni with N deprivation medium except the root length. Interestingly, Ni with P and K deprivation mediums had similar impact on root fresh biomass compared with plants grown on normal N, P, and K medium. Further, they probed that Ni alone in combination with N, P, and K deprivation induced stresses caused the generation of ROS, lipid peroxidation and reduction in antioxidant machinery except GR and vitamin E. Also, they recorded that Ni stress caused the reduction of N in shoot and in reverse N deprivation plants stored more Ni content in shoot. However, seedlings with K deprivation stored more Ni in root. Lastly, they also noted the positive impact of seed priming with selenium (Se) and salicylic acid (SA) under Ni stress or nutrient deprivation condition. As expected, their findings showed that Se and SA primed seedlings expressed more tolerance to Ni and no nutrient environment and better seedling growth and fresh biomass, which may be due to lower ROS level, greater membrane stability, improved antioxidant machinery and nutrient homeostasis.

The term “hyper accumulator” was first time used by Isnard et al. in their report published in 1976. According to their finding and later on observations of other researchers, hyper-accumulator are those plants that accumulate specific metal or metalloids in their tissues (living) 100 or 1,000 times more than normal uptake for most of plant species. In line with above, Isnard et al. conduct a comprehensive study hydroponically to explore the genetic mechanism in Ni hyperaccumulation and then its translocation in genus *Pycnandra*. Their data (after foliar application of Ni) stated that the genus *Pycnandra*, species acuminata can resist up to 3,000 μM. Moreover, the Ni portioning was spread throughout the plant body, but was more obvious in latex (124,000 μg g−1). Further, their phylogenetic analysis revealed that phenomenon of “hyperaccumulation” evolved independently in two subgenera and five species of the above-mentioned genus. Later on, extreme resistance to Ni stress becomes the unique property of lacticifers. As a whole, these findings suggested further investigation to explore the genetic makeup of latex producing cells to explore their Ni storing mechanism/s.

Now a days researchers are more anxious about probing the consequences of HMs starts from the external environment *via* their uptake or bioaccumulation into different parts of plant's body. There is such example of engineered nanoparticles of Ni oxide (NiO-NP) investigated by Manna et al. in the roots of *Allium cepa*. Their results showed that before entering into cell organelles, NiO-NP first damage the cell membrane and then disturb the cellular homeostasis and finally minimize the survival chances. In result, a boom in the generation of ROS and nitric oxide (NO) were occurred resulted in the alteration of several important biochemicals. Meanwhile, in response to external stress, transcription rate and activities of antioxidants such as CAT, POD, SOD were enhanced (on an average up to 50–250%). Moreover, their results showed that the transcript levels of two subunits of Rubisco activase were also increased. Besides, they noticed that increased in the NO content was dose dependent and also may due to increase in the accumulation of other enzymes such as NADPH oxidase, NO synthase and nitrate reductase. Moreover, they also highlighted the general cellular response (changes in ROS-NO nexus) to external stress, for instance, modifications in K/Na ratio, enhancement in proline, and GABA content. Overall, above findings not only explored the detailed negative consequences of NiO-NP but also highlighted the response of general stress indicators.

## Al-Induced Toxicity in Plants: Impact on PG&D, ROS and Antioxidant Machinery, and Its Alleviation Through Over-Expression of Related Gene/s

Soils having pH lower than ≤ 5.5 considered as acid soils. In total, acid soils comprise of 30% of arable and 50% of potential cultivated land. Al is the third most abundant element present in the earth crust that can be converted to soluble and lethal form (Al^+3^), which is the promising limiting factor of PG&D in acid soils that ultimately impact on seed yield. Du et al. explored the genes participate in Al tolerance mechanism by taking maize crop plant as an example. His results showed that *aluminum tolerance 6* (*ZmAT6*) gene that expressed in almost all tissue/organs but upregulated more in shoot and then root under Al stress. Further, investigation in the over-expressed maize and Arabidopsis plants confirmed the role of *ZmAT6 in* Al tolerance as in the transgenic plants better root growth, lower Evans blue absorption, lower accumulation of Al, reduction in the generation of ROS, increment in the proline content and several antioxidants such as SOD (including its transcript level, *ZmSOD*), POD, CAT, and APX were recorded compared to WT.

## The Alleviating Role of H_2_S in Plants Under HM Stress

Lastly, Arif et al. presented the perspective on the role of H_2_S in alleviating the negative impacts of HMs in plants. They discuss the detailed role of H_2_S as a signaling molecule counter the HMs induced ROS and then improves plant tolerance. In detail, the alleviating role of H_2_S in terms of eliminating the negative impacts of metal-element induced toxicity, improving several key physiological and biochemical processes. Lastly, they argued on the cross-talk of H_2_S with other endogenous hormones improves plant growth and development and mitigated the metal induced phytotoxicity under hazardous environmental conditions.

## Author Contributions

RG and BA wrote the first draft of the article. MK and AR made a substantial, intellectual contribution to the work, and approved it for publication. All authors contributed to the article and approved the submitted version.

## Conflict of Interest

The authors declare that the research was conducted in the absence of any commercial or financial relationships that could be construed as a potential conflict of interest.

## Publisher's Note

All claims expressed in this article are solely those of the authors and do not necessarily represent those of their affiliated organizations, or those of the publisher, the editors and the reviewers. Any product that may be evaluated in this article, or claim that may be made by its manufacturer, is not guaranteed or endorsed by the publisher.

